# Harnessing Gut Microbiota for Biomimetic Innovations in Health and Biotechnology

**DOI:** 10.3390/biomimetics10020073

**Published:** 2025-01-24

**Authors:** Ana Isabel Beltrán-Velasco, Vicente Javier Clemente-Suárez

**Affiliations:** 1 NBC Group, Psychology Department, School of Life and Nature Sciences, Nebrija University, 28248 Madrid, Spain; 2 Faculty of Sports Sciences, Universidad Europea de Madrid, Tajo Street, s/n, 28670 Madrid, Spain; vctxente@yahoo.es; 3 Grupo de Investigación en Cultura, Educación y Sociedad, Universidad de la Costa, Barranquilla 080002, Colombia

**Keywords:** gut microbiota, biomimetics, personalized medicine, bioinspired therapies, artificial microbiomes, probiotics and synbiotics, microbiota–gut–brain axis, biosensors, intestinal health, microbiome innovation

## Abstract

The gut microbiota is a complex and dynamic ecosystem that plays a fundamental role in human health by regulating immunity, metabolism, and the gut–brain axis. Beyond its critical physiological functions, it has emerged as a rich source of inspiration for biomimetic innovations in healthcare and biotechnology. This review explores the transformative potential of microbiota-based biomimetics, focusing on key biological mechanisms such as resilience, self-regulation, and quorum sensing. These mechanisms have inspired the development of innovative applications, including personalized probiotics, synbiotics, artificial microbiomes, bioinspired biosensors, and bioremediation systems. Such technologies aim to emulate and optimize the intricate functions of microbial ecosystems, addressing challenges in healthcare and environmental sustainability. The integration of advanced technologies, such as artificial intelligence, bioengineering, and multi-omics approaches, has further accelerated the potential of microbiota biomimetics. These tools enable the development of precision therapies tailored to individual microbiota profiles, enhance the efficacy of diagnostic systems, and facilitate the design of environmentally sustainable solutions, such as waste-to-energy systems and bioremediation platforms. Emerging areas of innovation, including gut-on-chip models and synthetic biology, offer unprecedented opportunities for studying and applying microbiota principles in controlled environments. Despite these advancements, challenges remain. The replication of microbial complexity in artificial environments, ethical concerns regarding genetically engineered microorganisms, and equitable access to advanced therapies are critical hurdles that must be addressed. This review underscores the importance of interdisciplinary collaboration and public awareness in overcoming these barriers and ensuring the responsible development of microbiota-based solutions. By leveraging the principles of microbial ecosystems, microbiota biomimetics represents a promising frontier in healthcare and sustainability. This approach has the potential to revolutionize therapeutic strategies, redefine diagnostic tools, and address global challenges, paving the way for a more personalized, efficient, and sustainable future in medicine and biotechnology.

## 1. Introduction

### 1.1. The Importance of the Intestinal Microbiota in Human Health

The gut microbiota, comprising trillions of microorganisms, including bacteria, viruses, fungi, and archaea, constitutes a complex biological ecosystem intrinsically related to human health. This microbial assemblage contributes to the digestion and absorption of nutrients, as well as to the regulation of fundamental immune processes, protection against pathogens, and the synthesis of essential metabolites, such as short-chain fatty acids (SCFAs), neurotransmitters, and vitamins. The dynamic interplay between the microbiota and the intestinal epithelium is crucial for maintaining the integrity of the intestinal barrier, preventing bacterial translocation, and modulating the inflammatory response [1,2].

However, alterations in the composition or function of the intestinal microbiota, a phenomenon known as dysbiosis, have been linked to various pathologies, including inflammatory bowel diseases, type 2 diabetes, obesity, colorectal cancer, neuropsychiatric disorders such as depression and anxiety, neurodegenerative pathologies such as Alzheimer’s Disease or Parkinson’s Disease, and others [3]. Consequently, the microbiota has emerged as a pivotal therapeutic target in the realm of emerging biomedical research, propelling the development of interventions such as probiotics, synbiotics, fecal microbiota transplants (FMTs), and compounds derived from beneficial microorganisms (metabiotics) [4].

Probiotics are defined as live microorganisms that, when administered in adequate amounts, confer health benefits to the host by balancing the microbiota diversity and reinforcing its functions [5]. Conversely, synbiotics are defined as the combination of probiotics and prebiotics (non-digestible substrates that promote the growth of beneficial bacteria) to enhance their therapeutic effects [6]. A further classification of probiotics is that of psychobiotics, which are bacterial strains that have a direct impact on the host’s mental health and emotional well-being [7]. These act through the gut–brain axis, modulating the activity of the nervous system (NS) and the production of neurotransmitters, such as serotonin, to influence mood, anxiety, and other psychiatric disturbances [8]. A significant illustration of this influence is butyrate, a metabolite that exerts neuroprotective effects by modulating the expression of genes implicated in neuronal plasticity and neuroinflammation. Moreover, butyrate regulates the permeability of the blood–brain barrier, thereby enabling communication between the gut and the brain [9]. This growing interest underscores the necessity to explore how the functional principles of this ecosystem can inspire technological innovations in healthcare.

### 1.2. Concept of Biomimetics: Definition and Relevance to Medical Care

Biomimetics, defined as the study and application of biological principles to solve human problems, has emerged as a promising interdisciplinary approach integrating biology, engineering, medicine, and material sciences [10]. Drawing inspiration from the evolutionary and adaptive processes of nature, this field seeks to develop sustainable and effective solutions that emulate the strategies of living organisms. Examples include the design of self-repairing materials based on the structure of bone tissue, the creation of adhesives inspired by octopus suction cups, and the development of computational algorithms based on swarming behavior patterns [11].

In the field of healthcare, biomimetics offers unique opportunities to address complex challenges such as antimicrobial resistance, regenerative medicine, and advanced diagnostic systems. In this regard, the gut microbiota represents a paradigmatic example of how a biological ecosystem can inspire innovations [12,13]. The mechanisms of intercellular communication (*quorum sensing*), ecological adaptation, and the production of bioactive metabolites in the gut microbiota have been employed as models for the development of biosensors, targeted therapies, and bioartificial systems [14,15]. As technological advancements in the field of microbiome intervention continue to evolve, it becomes imperative to elucidate the mechanistic processes that enable alterations in the microbiota to modulate host biology at the molecular level. These underlying mechanisms are paramount for the development of effective, personalized microbiome-based therapeutic interventions.

The present article aims to provide a comprehensive analysis of the contributions of the gut microbiota to the field of biomimetics applied to healthcare, with a particular focus on its applications in the development of innovative therapies and technologies. A narrative review of the most recent literature will be conducted to address three fundamental aspects: (1) the key biological mechanisms of the microbiota that have served as biomimetic inspiration; (2) the technological advances derived from these ideas, including probiotic-based therapies, synbiotics, and bioartificial systems; and (3) the current barriers and future opportunities in the integration of bioinspired principles into clinical practice. This approach will allow for the compilation of the most significant data from this field of study, as well as the provision of a forward-looking view on the transformative potential of biomimetics in healthcare, particularly in harnessing the functional principles of the gut microbiota.

## 2. Materials and Methods

The present narrative review aims to explore the gut microbiota as a source of biomimetic inspiration for innovations and applications in healthcare. A critical approach was applied to identify, analyze, and synthesize the most relevant advances on the subject, based on original studies, systematic reviews, and narratives of interest that address the relationship between the gut microbiota and its biomimetic applications. For the selection of studies, inclusion and exclusion criteria were established to ensure the relevance and quality of the articles.

The inclusion criteria were met by studies that examined the role of the intestinal microbiota in biomimetic applications in medicine, as well as studies that proposed technological innovations based on the functioning of the intestinal microbiota. Exclusion criteria were met by studies that did not address the biomimetic approach and studies that did not link the gut microbiota to medical care. A comprehensive search of several widely accepted scientific databases, including PubMed, Scopus, Web of Science, Wiley, and ScienceDirect, was conducted to ensure exhaustive coverage. The search was limited to studies published between 2010 and 2025, with a particular emphasis on those that explored innovative aspects related to biomimetics. The search terms employed included combinations of keywords such as “gut microbiota”, “biomimetic applications”, “medical innovations”, “biomimetics in healthcare”, and “microbiota-based technologies”. The application of Boolean operators was utilized to optimize the search and to encompass a comprehensive array of biomimetic applications related to gut microbiota. Following the implementation of these filters and the selection of studies according to the established criteria, a review of the titles and abstracts was conducted. The studies that met the inclusion criteria were then subjected to a thorough review. To ensure objectivity and minimize potential biases, the selection of articles was carried out independently by two reviewers.

## 3. Gut Microbiota: A Complex Biological Ecosystem

In recent years, the study of the intestinal microbiota has revealed its influence on various aspects of human health, thereby establishing its relevance in modern medicine [16]. In particular, the analysis of its functions and its interaction with the host has opened new perspectives for the creation of biomimetic applications. The objective of these applications is to emulate the functional principles of this microbial ecosystem to develop therapeutic and technological innovations in medical care [17].

### 3.1. Composition and Main Functions of the Gut Microbiota

The human intestinal microbiota is predominantly bacterial in nature, though it also encompasses viruses, fungi, and archaea. This microbial community is distributed along the gastrointestinal tract, with a density observed in the colon. The composition of the microbiota exhibits interindividual variability, influenced by genetic factors, dietary habits, environmental influences, and exposure to antibiotics, among other factors. The predominant bacterial phyla include *Bacillota* (formerly *Firmicutes*), *Bacteroidetes*, *Actinobacteria*, and *Proteobacteria*, which play pivotal roles in the function of the microbiome [18,19,20].

*Bacillota*, a dominant phylum within the intestinal microbiome, encompasses a wide array of genera that play pivotal roles in intestinal metabolism and homeostasis. Noteworthy genera include *Lactobacillus* and *Enterococcus*, which are distinguished by their fermentative capabilities and the production of lactic acid. This acid contributes to the regulation of intestinal pH and the inhibition of the growth of potentially harmful microorganisms. Other genera, such as *Faecalibacterium*, *Ruminococcus*, and *Clostridium*, play an essential role in the degradation of complex polysaccharides and in the production of short-chain fatty acids. Within the *Bacillota* phylum, *Faecalibacterium prausnitzii* has been identified as a marker of intestinal health due to its anti-inflammatory activity and contribution to intestinal barrier integrity [21]. The phylum *Bacteroidetes*, comprising the genera *Bacteroides* and *Prevotella*, plays a pivotal role in the degradation of complex carbohydrates and the production of bioactive metabolites that influence host homeostasis. Notably, *Bacteroides fragilis* has been characterized as a regulator of the immune system due to its capacity to modulate the inflammatory response through the production of capsular polysaccharides, which promote the induction of regulatory T cells. The relative proportions of *Bacteroides* and *Prevotella* in the human intestine have been associated with specific dietary patterns, with a predominance of *Bacteroides* in Western diets rich in fats and animal proteins, while *Prevotella* is more abundant in individuals whose diet is rich in fiber and complex carbohydrates [22,23].

Despite comprising a smaller proportion of the intestinal microbiota, the Actinobacteria phylum plays a pivotal role in the gastrointestinal tract. Genera such as Bifidobacterium, which are predominant within this phylum, have been identified as crucial in the fermentation of oligosaccharides and the subsequent production of metabolites that have been shown to have a beneficial impact on both the integrity of the intestinal barrier and the immune system. Specifically, *Bifidobacterium longum*, *Bifidobacterium breve*, and *Bifidobacterium adolescentis* species are of particular significance in the colonization of the intestine during the initial years of life and have been associated with probiotic effects and benefits for intestinal health [24,25].

*Proteobacteria*, while present in low proportions in healthy individuals, have been linked to inflammatory states and alterations in intestinal homeostasis. This phylum encompasses genera such as *Escherichia*, *Klebsiella*, and *Salmonella*, which can act as opportunistic pathogens under conditions of dysbiosis. The presence of certain commensal strains of *Escherichia coli* in the human intestine is considered normal and contributes to vitamin synthesis and the regulation of the microbiome. However, the overgrowth of these strains or the proliferation of pathogenic strains can trigger inflammatory processes and intestinal diseases [26].

Other bacterial phyla that are less abundant but functionally relevant include *Verrucomicrobia* and *Fusobacteria*. Within *Verrucomicrobia*, *Akkermansia muciniphila* has been described as a key microorganism in the degradation of intestinal mucus and in the regulation of epithelial homeostasis, playing a potentially beneficial role in the integrity of the intestinal barrier and in the modulation of energy metabolism. Conversely, the genus *Fusobacterium nucleatum*, classified within the family *Fusobacteriaceae*, has been observed to be associated with immunomodulatory functions and various pathological states, including inflammatory diseases and specific forms of colorectal cancer.

The intestinal microbiota plays a multifaceted role in host health, extending beyond basic digestive functions. A pivotal function of the microbiota is the fermentation of non-digestible carbohydrates, which leads to the production of SCFAs. These SCFAs exert substantial effects on various physiological processes, including metabolic regulation, immune function, and intestinal health [27,28]. The gut microbiota also synthesizes essential vitamins, including B-complex vitamins (B6, B12, folate) and vitamin K, which are crucial for cell metabolism, red blood cell formation, and blood clotting. Furthermore, the microbiota plays a pivotal role in protecting against pathogens through competition for nutrients and the production of antimicrobial compounds. Various bacterial species produce substances such as bacteriocins, which inhibit the growth of pathogenic microorganisms, thereby helping to maintain microbial balance and prevent infections. The intestinal microbiota plays an active role in the maturation of the immune system, modulating immune tolerance and defense against pathogens. This contributes to the regulation of inflammation and the prevention of autoimmune diseases, making this balance crucial to avoid inappropriate immune responses that could lead to disorders such as inflammatory bowel disease (IBD) or allergies [29,30].

### 3.2. Microbiota–Host Interaction: Immune Regulation, Metabolism, and Communication

The relationship between the intestinal microbiota and the host is bidirectional, meaning that not only do intestinal microorganisms affect the host, but also the host environment modulates the composition and activity of the microbiota. One of the most prominent aspects of this interaction is immune regulation, as the gut microbiota influences the maturation and functioning of the immune system [31]. Intestinal bacteria communicate with immune cells through molecular signals, such as cytokines, modulating the immune response and contributing to homeostasis [31,32].

The most significant role of the gut microbiota is its capacity to modulate the host immune system, contributing to its development, maintaining balance, and preventing inappropriate immune responses [33]. Intestinal bacteria interact with immune system cells through a variety of molecular mechanisms, including the production of molecular signals such as cytokines and pathogen-associated molecular patterns (PAMPs), which are critical in the activation and regulation of immune responses [34,35].

Interactions between microbiota and immune cells primarily occur at the level of intestinal epithelial cells, which act as a protective barrier [36]. These cells can release immune signals that activate lymphocytes and other immune system cells, thereby promoting a response that maintains homeostasis. The production of anti-inflammatory cytokines, such as IL-10, stimulated by intestinal microorganisms, plays a crucial role in regulating chronic inflammation. At the molecular level, SCFAs have been shown to activate receptors on immune cells in the intestine, such as Toll-like receptors (TLRs), thereby modulating the immune response. This mechanism has direct implications for the regulation of inflammation and the immune response to pathogens [37]. Thus, *Faecalibacterium prausnitzii*, one of the most abundant bacteria in a healthy intestine, is known for its anti-inflammatory effect by stimulating the production of interleukin-10. In a similar manner, *Bacteroides fragilis* produces capsular polysaccharides that possess immunomodulatory capacity, thereby favoring the induction of regulatory T cells that control the inflammatory response [38,39]. However, the presence of dysbiosis, or an imbalance in the composition of intestinal microbiota, can favor an increase in inflammatory responses, which is related to various autoimmune and inflammatory diseases [40,41].

The gut microbiota also has a direct impact on host metabolism, as gut microorganisms are capable of breaking down complex food compounds that cannot be digested by human enzymes, such as certain types of fiber and non-absorbable carbohydrates [42]. This breakdown results in the production of key metabolites, such as SCFAs, which are products of bacterial fermentation [43]. SCFAs, including acetate, propionate, and butyrate, are absorbed by intestinal epithelial cells and utilized as a source of energy, thereby contributing to intestinal health and the prevention of inflammation [44,45]. The synthesis of these metabolites is facilitated by the presence of different bacterial species, each of which plays a distinct role. For instance, *Roseburia intestinalis* and *Eubacterium rectale* have been observed to be efficient producers of butyrate, a substance that exhibits anti-inflammatory properties [46].

Conversely, these metabolites can exert systemic effects, influencing the overall energy homeostasis of the organism. SCFAs have been associated with the regulation of glucose and adiposity, improving insulin sensitivity and modulating fat accumulation in adipose tissue [47]. The gut microbiota exerts a pivotal influence on glucose and lipid metabolism, thereby impacting body weight, the prevention of obesity, and fat metabolism. This underscores a direct association between the microbiota and metabolic diseases, including type 2 diabetes and metabolic syndrome [48,49]. In this sense, *Bacteroides thetaiotaomicron* contributes to the production of propionate, a substance that influences the regulation of lipid metabolism and glucose homeostasis [50].

Beyond SCFAs, the microbiota also produces bioactive metabolites, including indoles, which may impact liver function and the detoxification of potentially harmful compounds [51]. Indoles, which are derived from tryptophan metabolism by species such as *Clostridium sporogenes* and *Lactobacillus reuteri*, have been shown to play a role in modulating intestinal barrier and hepatic homeostasis [52,53]. Additionally, neurotransmitters such as serotonin, which influence both the central nervous system (CNS) and the regulation of various metabolic processes, are also generated by the microbiota [54]. This suggests that the gut microbiota exerts a multifaceted influence on both local digestive metabolism and overall body homeostasis [55,56]. It has been demonstrated that *Escherichia coli* and *Bifidobacterium adolescentis* play a role in the production of neurotransmitters, including serotonin and dopamine [57,58].

In recent years, significant attention has been directed toward the study of interactions between the gut microbiota and the CNS, collectively referred to as the microbiota–gut–brain axis [59]. This axis encompasses the transmission of chemical and nerve signals between the gastrointestinal tract and the brain, thereby enabling gut microorganisms to exert influence on the host’s behavior, mood, and cognitive function [60]. The microbiota employs diverse mechanisms to exert its influence, including the release of chemical signals such as neurotransmitters and hormones, as well as the stimulation of the vagus nerve, which functions as a direct conduit between the gut and the brain [61].

Recent research has demonstrated the capacity of the gut microbiota to influence the production of key neurotransmitters, including serotonin and dopamine, which are imperative for emotional well-being and mood regulation [62,63]. It is estimated that 90% of the body’s serotonin is located in the gut, thereby underscoring the significance of the gut microbiota in regulating brain function and mental health [64,65].

This interaction is also linked to neurological and psychiatric disorders [66,67]. The presence of dysbiosis, or an imbalance in gut microbiota, has been associated with autism spectrum disorders, psychotic disorders, and neurodegenerative pathologies, such as Parkinson’s Disease and Alzheimer’s Disease [68]. The activation of the vagus nerve by microbial metabolites and the production of intestinal neuromodulators, such as γ-aminobutyric acid (GABA) by *Lactobacillus rhamnosus*, have been proposed as key mechanisms in this interaction [69]. An imbalance in gut bacteria can trigger an increase in systemic inflammation, which can affect brain function and potentially contribute to the development of these disorders. Current psychobiotic studies are exploring the potential of manipulating the gut microbiota to improve emotional well-being and address neuropsychiatric disorders [70,71].

### 3.3. Production of Key Metabolites: Short-Chain Fatty Acids, Indoles, and Neurotransmitters

The gut microbiota plays a crucial role in the production of bioactive metabolites that have significant effects on host physiology. Among these metabolites, SCFAs, indoles, and neurotransmitters are some of the most relevant, as they affect various biological functions, from intestinal health to metabolism and brain function [72,73].

SCFAs play a pivotal role in regulating intestinal homeostasis. These metabolites serve as an energy source for intestinal epithelial cells and are critical in maintaining the integrity of the intestinal barrier [74]. They protect against pathogens and modulate both local and systemic inflammation. Furthermore, SCFAs have regulatory effects on global metabolism, influencing insulin sensitivity, fat storage, and control of glucose levels [75,76]. Therefore, SCFAs are produced by the intestinal microbiota and interact with short-chain fatty acid receptors (FFAR2) on intestinal epithelial cells. This interaction activates signaling pathways that promote the expression of genes involved in glucose regulation. Consequently, this contributes to improved insulin sensitivity in the host. *Eubacterium rectale* and *Roseburia intestinalis* are among the primary butyrate-producing species. Conversely, propionate, produced by bacteria such as *Bacteroides thetaiotaomicron* and *Veillonella* spp., plays a pivotal role in the regulation of glucose metabolism. Acetate is synthesized by various species, including *Bifidobacterium* and *Prevotella* spp. [77].

Indoles, which are derived from the amino acid tryptophan, are also pivotal metabolites that are produced by the gut microbiota [78]. These compounds exert effects at both the local and systemic levels, given their involvement in the regulation of intestinal function, the modulation of the immune response, and the protection against oxidative stress. Additionally, indoles influence the regulation of lipid metabolism and may have implications for metabolic diseases such as obesity [79]. Thus, bacteria such as *Clostridium sporogenes* and *Lactobacillus reuteri* are responsible for the conversion of tryptophan into indole and its derivatives [80]. A number of indole derivatives have been shown to possess neuroprotective properties, with this effect being attributed to their antioxidant capacity. Other indole derivatives, including indole-3-propionic acid, have been observed to modulate the immune response at the intestinal level through the activation of the aryl hydrocarbon receptor (AhR) [81].

The gut microbiota contributes significantly to the production of neurotransmitters which play crucial roles in regulating intestinal motility and maintaining gut homeostasis. Locally, these neurotransmitters modulate gastrointestinal functions, but their influence extends beyond the intestine through the microbiota–gut–brain axis. This bidirectional communication system enables neurotransmitters to impact central nervous system activities, thereby affecting mood, behavior, and cognitive functions. Notably, approximately 90% of serotonin in the human body is synthesized within the gut, highlighting the critical role of the intestinal microbiota in neurochemical regulation. Recent research has identified *Candida tropicalis* and *Morganella morganii* as serotonin producers [82]. *Akkermansia muciniphila* and *Parabacteroides distasonis* have been shown to synthesize γ-aminobutyric acid, and *Klebsiella pneumoniae* and *Proteus vulgaris* have been described as capable of producing dopamine [83,84].

**Figure 1:**
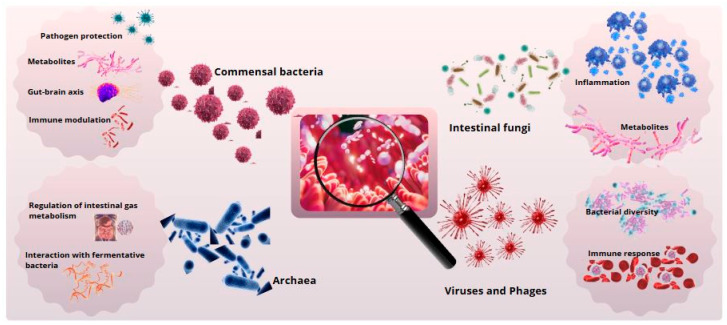
Gut microbial diversity and its biological functions. Primary functions of intestinal microbial groups. Commensal bacteria support digestion and immunity, archaea contribute to metabolism, fungi degrade polysaccharides, and viruses and phages regulate the microbiota.

As shown in Figure 1, such interactions underscore the profound implications of the gut microbiota in the pathophysiology of neurological and psychiatric disorders, including depression, anxiety, and neurodegenerative diseases. Advances in psychobiotics—probiotics targeting mental health—offer promising therapeutic avenues by leveraging the microbiota’s capacity to influence neurochemical pathways and emotional well-being [63].

## 4. Biomimetics Applied to Gut Microbiota

Biomimetics, or biomimicry, refers to the practice of mimicking the processes, structures, and functions of biological systems to solve human problems in various disciplines [85]. In this context, the gut microbiota has emerged as an invaluable source of inspiration for the development of biomimetic solutions in relevant fields such as medicine or biotechnology, among others. Biological principles derived from the gut microbiota, such as resilience, self-regulation, and microbial communication, offer innovative perspectives for the creation of advanced technologies that mimic these natural mechanisms [86,87,88].

### 4.1. Bioinspired Concepts Derived from the Microbiota

#### 4.1.1. Resilience and Self-Regulation of Microbial Ecosystems

One of the fundamental principles derived from the gut microbiota that offers enormous potential for biomimetics is the resilience and self-regulatory capacity of microbial ecosystems [89]. The gut microbiota, as a complex biological ecosystem, exhibits a remarkable capacity to adapt to variations in its internal and external environment and to maintain its functional balance despite perturbations. This capacity for resilience, which is essential for maintaining host homeostasis, serves as a paradigm for how biological systems can resist and adapt to change, thereby ensuring their stability and functionality over time [90,91].

The intestinal microbiota demonstrates resilience in several ways. Alterations in the composition of microbial communities, induced by external changes such as a shift in diet or the use of antibiotics, prompt the system to reorganize and reestablish a functional microbial structure [92,93]. However, these alterations in composition often result in a new equilibrium, a phenomenon referred to as recovery [94]. This process involves the ability of bacteria and other microorganisms to rebalance their metabolic functions, as well as the ability of host cells to adjust their immune responses and interactions with the microbiota. Even when the microbiota is affected by pathologies or dysbiosis, the system has mechanisms to induce the restoration of balance and the optimization of its long-term function [95,96].

The concept of self-regulation in the intestinal microbiota is equally pertinent. The intestinal microbiota is a dynamic system that is in constant adaptation to environmental changes while also interacting with each other and the host [97]. This intricate relation is coordinated by processes such as *quorum sensing*, which allows bacteria to communicate via chemical signals, coordinating their activities and adjusting their population in a way that maintains ecological stability [98]. This phenomenon exemplifies a form of self-regulation, wherein microbial populations adapt to fluctuating environmental conditions, thereby ensuring a dynamic equilibrium among the species present [99,100].

The principle of resilience and self-regulation has provided the inspiration for a series of biomimetic innovations, especially in the design of artificial systems that seek to imitate the adaptive capacity of microbial ecosystems [101]. In the field of engineering and biotechnology, bioreactors and cell culture devices have been developed that replicate the ability of the microbiota to adjust its metabolic activity and respond to external stimuli in a flexible manner [102]. These biomimetic systems are capable of maintaining optimal conditions for growth and functionality, even when faced with drastic changes in their operating conditions [103].

In biomimetic applications within medicine, the resilience and self-regulation of the microbiota can be applied to the creation of technologies that promote homeostasis and recovery in human health contexts [104]. An example of this is the design of smart prostheses that are able to adapt to changing conditions in the body, such as fluctuations in pH or temperature. Furthermore, the utilization of biomimetic principles in the domain of personalized microbiological therapies, such as probiotics or FMT, holds considerable potential to enhance therapeutic efficacy by tailoring microbial interactions and their impact on the host according to the specific needs of each patient [105,106].

#### 4.1.2. Microbial Intercellular Communication (Quorum Sensing)

*Quorum sensing* is a sophisticated form of microbial intercellular communication that enables bacteria and other microorganisms to coordinate their collective behaviors through chemical signaling. This mechanism is density-dependent, allowing microbial communities to regulate essential biological processes such as biofilm formation, production of metabolites, expression of virulence factors, and modulation of host immune responses [86,87]. These coordinated behaviors are crucial for maintaining microbial ecosystem stability and host–microbiota interactions, especially in dynamic environments like the intestinal tract.

In the gut microbiota, *quorum sensing* plays a central role in regulating microbial interactions and maintaining ecological balance. Through signaling molecules such as autoinducers, bacteria can detect population density and adjust their activities accordingly, optimizing colonization, resource utilization, and defense mechanisms [88,89]. For instance, *quorum sensing* controls the production of antimicrobial peptides that inhibit the overgrowth of pathogens while fostering the development of protective biofilms that shield beneficial communities from environmental stressors such as pH changes and antibiotic exposure [90,91]. These biofilms are dynamic structures that not only ensure microbial resilience but also enhance nutrient exchange and metabolite production [92].

Beyond its role in microbial regulation, *quorum sensing* significantly impacts host health by influencing immune responses and gut homeostasis. Microbial signals can activate specific immune pathways, modulating inflammation and enhancing the gut barrier’s integrity. This regulatory effect is particularly important in preventing inflammatory conditions such as Crohn’s disease and ulcerative colitis, where dysregulated signaling can exacerbate pathology [93,94]. Moreover, *quorum sensing* has been implicated in the modulation of systemic processes, as microbial metabolites generated through quorum-regulated pathways can influence distant organs, including the brain, via the microbiota–gut–brain axis.

From a biomimetic perspective, *quorum sensing* has inspired the development of cutting-edge technologies that replicate bacterial communication for therapeutic and diagnostic purposes. High-precision biosensors, modeled on *quorum sensing*, are being designed to detect environmental changes and adjust their functionality in real time. These devices are applied in monitoring intestinal health, regulating the production of bioactive compounds, and optimizing microbial therapies [95,96]. For example, synthetic bacteria equipped with engineered *quorum sensing* circuits can be programmed to release therapeutic agents such as anti-inflammatory peptides or targeted antimicrobials when specific conditions, like pH or metabolite levels, are detected in the gut [97,98]. These advances offer promising avenues for treating conditions such as dysbiosis, infections, and chronic inflammatory diseases while minimizing unintended disruptions to the native microbiota [99,100].

In addition to clinical applications, *quorum-sensing*-inspired systems are being explored in industrial and environmental biotechnology. Microbial consortia with engineered *quorum sensing* capabilities are being developed to optimize bioreactors for pharmaceutical production or bioremediation processes. These systems emulate the adaptability and efficiency of natural microbial ecosystems, opening new frontiers for sustainable innovations.

### 4.2. Simulate Microbial Ecosystems in Artificial Environments

The simulation of artificial microbial ecosystems has emerged as a key tool for understanding the dynamics of bacterial communities and their collective behavior in controlled environments. Through the use of computational models and in vitro systems, it is possible to replicate phenomena such as competition, cooperation, and the adaptation of microbes to environmental changes [107]. This approach involves the design of systems that emulate the complexity and functionality of natural microbial ecosystems, with the objective of replicating their biological benefits in a controlled environment [108]. Through the engineering of synthetic or modified microbiotas, researchers can develop artificial models that replicate the interactions between microorganisms and their environment, enabling the study and utilization of these processes in practical applications [109].

In the field of healthcare, the simulation of microbial ecosystems holds promise for designing personalized treatments that target the modulation of the patient’s intestinal microbiota. The creation of artificial microbiotas through the combination of specific probiotic bacteria and prebiotics has the potential to offer solutions for the treatment of metabolic diseases, digestive disorders, and even neurological disorders associated with the microbiota–gut–brain axis. A critical element in the simulation of these ecosystems is the replication of microbial intercellular communication, particularly the phenomenon of *quorum sensing*. This mechanism enables bacteria to coordinate their activity according to cell density, a pivotal factor in processes such as biofilm formation and metabolite production. By modeling this behavior under artificial conditions, complex interactions and their impact on the stability and functionality of microbial communities can be investigated [110].

Additionally, advancements in the field of microbial ecosystem simulation have the potential to influence other areas of biotechnology, such as the production of bioactive compounds, bioremediation, and the development of new biocompatible materials. This is made possible by the creation of artificial environments containing genetically modified microorganisms [111,112].

## 5. Innovations Inspired by the Intestinal Microbiota

Recent advancements in the field of healthcare and biotechnology have been influenced by innovations inspired by the gut microbiota. The potential of the gut microbiota as a biomimetic model has led to the development of new therapies, technologies, and devices that emulate its biological functions. These developments aim to solve medical challenges and improve existing treatments [113].

### 5.1. Therapies Based on Probiotics and Synbiotics

#### 5.1.1. The Development of Probiotics Inspired by Biological Processes

Probiotics, which have been demonstrated to confer health benefits to the host, have been widely used for the treatment of gastrointestinal disorders and the improvement of intestinal health [114,115]. However, the concept of bioinspired probiotics extends beyond the mere administration of bacterial strains [116]. This novel approach involves the development of bacterial strains that emulate specific functions of the balanced intestinal microbiota. These functions include modulation of the immune response, production of essential metabolites (e.g., SCFAs), and protection against pathogens. These bioinspired probiotics can be designed to replicate the functional diversity and self-regulatory capacity observed in natural microbial ecosystems [117].

Advances in biotechnology and genetic engineering have enabled the precise modification of bacterial strains to execute specific therapeutic functions, ranging from regulating metabolism to safeguarding the NS [118]. The potential applications of bioinspired probiotics extend beyond the realm of digestive disorders, encompassing metabolic diseases, neurological disorders, and autoimmune diseases [119]. These probiotics leverage the intrinsic functions of the microbiota, aiming to restore the host’s biological equilibrium [120].

#### 5.1.2. Synbiotics Designed to Replicate Specific Functions of the Microbiota

Synbiotics are combinations of probiotics and prebiotics that, when administered in conjunction, promote a healthy balance of the intestinal microbiota [121]. This biomimetic innovation, inspired by the gut microbiota’s ability to maintain homeostasis, has garnered significant attention in the field of biomedical research [121,122]. Synbiotics provide beneficial microorganisms (probiotics) and non-digestible substrates (prebiotics), which stimulate the growth and activity of these microorganisms. This combination is designed to more effectively replicate the functions performed by the natural intestinal microbiota in the body, such as the digestion of nutrients, the modulation of the immune response, and the production of beneficial metabolites [123].

The field of biomimetic research has identified the design of personalized synbiotics as one of its most promising areas of interest and growth. The unique composition of each individual’s gut microbiota necessitates the tailoring of synbiotics to meet the specific needs of each person, taking into account factors such as the composition of their microbiota, their diet, and their health status [124]. This customization represents a significant step towards more precise and effective therapies, with an individualized approach that can optimize intestinal function and improve the overall health of the host [125,126].

In the context of inflammatory bowel diseases such as Crohn’s disease or ulcerative colitis, synbiotics, which are designed to replicate the functions of the intestinal microbiota, have been shown to help restore altered microbial balance, thereby reducing intestinal inflammation and enhancing immune response [127,128,129]. In the context of metabolic disorders, such as obesity or type 2 diabetes, synbiotics have been shown to promote the production of SCFAs, which are critical for regulating metabolism and systemic inflammation. By supplying beneficial microorganisms and suitable prebiotics for their proliferation and activity, these products can enhance the metabolic and anti-inflammatory benefits derived from a balanced gut microbiota [130,131].

Furthermore, the potential for synbiotics to contribute to disease prevention is noteworthy. Specifically, they have the capacity to preserve a healthy microbial balance in individuals susceptible to developing metabolic disorders or autoimmune diseases. Among other benefits, synbiotics could play a pivotal role in preventing dysbiosis before it escalates into a significant health concern, thereby promoting balanced immune function and efficient digestion from an early stage [132].

### 5.2. Artificial Microbiomes and Bioartificial Systems

#### 5.2.1. Artificial Models for Simulating Host–Microbiota Interactions

The development of artificial microbiomes has emerged as a revolutionary innovation in the field of biomimetics, providing a novel perspective for the study and manipulation of the intestinal microbiota [109]. These models facilitate the simulation, analysis, and comprehension of fundamental biological processes in a controlled and reproducible manner. Through these systems, the study of microbiota–host dynamics can occur without the limitations of traditional in vivo models, thereby opening a range of possibilities for both basic and applied research [133,134].

Artificial microbiomes are systems constructed using multi-species microbial cultures that mimic the biodiversity and functionality of the gut microbiota [135]. These models are designed to simulate the physiological conditions of the human intestine, allowing for direct observation of how microorganisms interact with each other and with host cells, how they modulate the immune response, and how they affect metabolic processes such as digestion, nutrient absorption, and energy regulation [136,137]. By establishing these controlled conditions, researchers can systematically manipulate variables such as microbial composition, pH, nutrient availability, and oxygen conditions. This approach facilitates the observation of the effects of different factors on the microbiota and their influence on health [138].

These artificial models permit detailed analysis of interactions between microorganisms and the host, in addition to offering a powerful tool for the development of new microbiota-based therapies [139]. One may test different combinations of bacterial strains, evaluating their ability to restore the balance of the microbiota in conditions of dysbiosis or their impact on metabolic and autoimmune diseases [140]. Furthermore, artificial microbiome models facilitate the study of therapeutic interventions, such as probiotics, prebiotics, or synbiotics, allowing the identification of more effective formulations before testing them in humans. This ability to perform trial and error in a controlled environment significantly reduces the risks associated with clinical interventions [141].

A particularly promising area of artificial microbiome development pertains to its application in the field of FMT (a therapeutic intervention in which intestinal microbiota is transferred from a healthy donor to a patient with severe intestinal dysbiosis). This emerging technique is used to restore the gut microbiota in patients with severe disorders, such as *Clostridioides difficile* infection or inflammatory bowel diseases [142]. However, the effectiveness of FMT may be affected by the heterogeneity of the donor microbiota and the recipient’s ability to integrate the transplanted bacteria [143]. The employment of artificial models offers a promising avenue for enhancing the efficacy and safety of FMT. These models facilitate the evaluation of diverse combinations and the simulation of various scenarios, thereby enabling the optimization of donor selection and treatment personalization. In addition, this ensures that FMT is more effective and less likely to induce adverse effects [144].

Beyond their clinical applications, artificial microbiomes are instrumental in elucidating the functions of the gut microbiota in pathological conditions. These models offer a controlled laboratory environment conducive to the study of complex phenomena, including chronic inflammatory responses, the impact of antibiotics, and the role of the microbiota in protection against pathogens [145,146]. The manipulation of microbiota within these systems enables researchers to identify novel biomarkers for diseases associated with intestinal dysbiosis and to develop novel strategies to restore the microbiota in pathological states.

#### 5.2.2. Applications in Fecal Microbiota Transplants

FMT has proven to be highly effective in the treatment of recurrent infections associated with prolonged use of antibiotics [147]. Beyond this context, FMT is being explored as a therapeutic tool in inflammatory bowel diseases, metabolic disorders, and even conditions related to the microbiota–gut–brain axis, such as neuropsychiatric and neurodegenerative disorders [148,149].

The integration of artificial microbiomes into the FMT process has the potential to transform its precision and efficacy. The development of bioartificial systems that replicate intestinal conditions enables preliminary assessment of the optimal microbial composition for each patient, thereby enhancing the selection of donors and facilitating personalization of the transplant by adjusting the microbial composition to the unique requirements of the recipient [150]. A significant advancement in this field is the capacity to forecast how diverse microbial combinations will interact with the host environment prior to transplantation. The employment of bioartificial systems facilitates the modeling of these interactions under controlled conditions, encompassing the immune response, nutrient metabolism, and the production of critical metabolites [151,152]. This approach enables the identification of potential adverse effects and ensures more effective integration of the transplanted microbiota.

Furthermore, the utilization of artificial microbiomes holds promise in facilitating the generation of standardized and reproducible microbial mixtures. This approach has the potential to circumvent a significant limitation of FMT, namely the inherent variability of the donor microbiota. This development presents a promising opportunity for the development of therapies based on specific, selected, and laboratory-grown microbial consortia, which would enhance the safety and consistency of treatment [153].

The development of artificial-intelligence-based modeling and simulation technologies could also complement advances in FMT. These tools would facilitate the analysis of substantial data on microbial composition and host responses, enabling the prediction of transplant success and the design of highly personalized therapies [154,155]. Specifically, machine learning algorithms hold promise in identifying patterns within the microbiota of successful donors, thereby guiding the selection of ideal donors for diverse clinical conditions [156,157].

The integration of artificial microbiomes and bioinspired technologies in FMT signifies a substantial advancement, paving the way for more effective, personalized, and safe treatments. This integration will optimize the intervention, thereby expanding the potential scope of FMT to encompass a broader spectrum of diseases associated with dysbiosis and the intestinal microbiota [158].

### 5.3. Bioinspired Devices and Biosensors

The design of bioinspired devices and biosensors represents an emerging field in which the functional principles of the intestinal microbiota are translated into technological innovations. The basis of these innovations is the intrinsic capacity of the microbiota to detect, process, and respond to biological stimuli [159]. The goal of these systems is to replicate, in artificial environments, the microbial interactions that take place within the intestinal ecosystem [160,161]. This approach offers advanced tools for the diagnosis and monitoring of diseases, as well as for the personalization of medical treatments based on the functional patterns of the microbiota.

#### 5.3.1. Diagnostic Tools Based on Functional Patterns of the Microbiota

A significant area of development pertains to the creation of diagnostic instruments that emulate the functional interactions between the microbiota and the host. These instruments are designed to assess not only the taxonomic composition of microbial communities but also the metabolic and biochemical functions that emerge from these interactions. Alterations in the production of metabolites, such as SCFAs, indoles, or neurotransmitters, reflect specific states of health or disease [162]. These alterations can be detected earlier through bioinspired diagnostic devices. This functional approach enables us to surpass conventional diagnostic methods that rely on late clinical parameters, thereby facilitating earlier and more precise interventions.

#### 5.3.2. Sensors That Detect Microbial Metabolites in Real Time

Advancements in biosensor technology have enabled the development of devices capable of monitoring the metabolic activity of the microbiota in real time. These sensors, designed to detect the presence and concentration of key metabolites, mimic the microbiota’s ability to dynamically respond to changes in the host’s internal environment. Continuous monitoring of metabolites such as butyrate, propionate, or acetate offers valuable information about the state of the individual’s intestinal and metabolic health [163]. Furthermore, these sensors facilitate rapid identification of deviations in microbial compound production associated with pathological states, such as volatile organic compounds produced in intestinal infections [164].

The implementation of sensors in wearable or implantable devices holds significant implications for the personalization of medicine. These devices, by generating data in real time, enable continuous patient monitoring and allow therapeutic strategies to be adjusted more precisely and dynamically [165]. For instance, an implantable sensor that detects a decline in anti-inflammatory metabolite production could trigger a controlled release system of prebiotics or probiotics, aimed at reestablishing microbial balance. This integrative approach signifies a shift towards biomedical systems that not only detect imbalances but also proactively interact with the patient’s biological environment to restore homeostasis [166,167].

**Figure 2:**
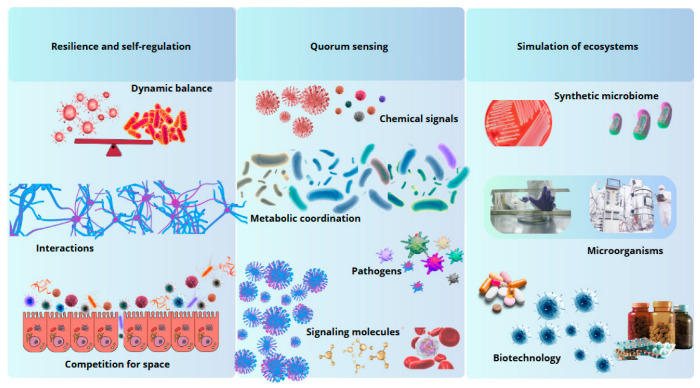
Biomimetic principles inspired by the intestinal microbiota. The gut microbiota offers models of resilience, intercellular communication, and ecosystem simulation, inspiring innovations in biotechnology and medicine.

As shown in Figure 2, in the academic and technological domain, bioinspired devices and biosensors exemplify an interdisciplinary convergence that integrates knowledge from microbiology, bioengineering, and biotechnology [168]. The development of these systems contributes to a greater understanding of the functional processes of the microbiota and marks a turning point in the design of medical technologies that seek to replicate and take advantage of the functional principles of biological systems [169,170]. This advancement underscores the potential of biomimetics as an innovative framework to transform both biomedical research and clinical practice.

## 6. Clinical Applications and Challenges

In recent years, microbial biomimetics has undergone significant advancements as a field of study. The development of personalized therapies based on microbiota offers novel opportunities to treat various pathologies, adapting treatments to the individual characteristics of each patient. This is achieved by analyzing the patient’s microbial profile, which can directly influence the body’s response to therapies, thereby optimizing treatment effectiveness.

The advent of sophisticated technologies, such as metagenomics, facilitates the precise evaluation of the microbial composition of individuals, thereby enabling the development of interventions aimed at modulating the gut microbiota and reestablishing its functionality. The personalization of therapeutic interventions may encompass the utilization of probiotics, prebiotics, fecal microbiota transplantation, and other microbial interventions, meticulously customized to address the distinct requirements of each patient. Furthermore, continuous monitoring through sequencing technologies and bioinformatics analysis is imperative to ensure the efficacy of therapeutic interventions and to make necessary adjustments to maximize therapeutic benefits.

## 7. Prospective Trends in Microbial Biomimetics

Emerging technologies, particularly artificial intelligence (AI), are playing a pivotal role in the advancement of microbiota-based therapies. AI can be delineated as the capacity of machines and computer systems to execute tasks that typically require human intelligence, such as learning, reasoning, decision-making, and natural language processing. AI enables the modeling and prediction of how microbiological interventions affect the organism, thereby facilitating the design of more targeted and personalized treatments. The analysis of extensive microbiological datasets facilitates the identification of patterns and correlations that would otherwise remain imperceptible, thereby contributing to the development of more efficacious therapeutic approaches.

Another salient trend is bioengineering, which further advances the development of microbiota therapies. Advances in bioengineering enable the modification and design of microorganisms to enhance their therapeutic capacity, thereby opening up new avenues for the treatment of diseases and rendering microbiota-based interventions more effective.

As the understanding of the interaction between the microbiome and the host deepens, new ways to balance the microbiota through genetic manipulation of bacteria and the creation of combination therapies are being identified. The future of microbial biomimetics may also include the use of technologies such as CRISPR to specifically alter microbial composition to treat diseases associated with microbiota imbalance.

However, there are challenges to the implementation of these personalized therapies. The long-term effects of manipulating the microbiota must be carefully evaluated, as there may be risks associated with permanent changes in its composition or resistance of some microbial species to the interventions. In addition, the stability and sustainability of these therapies are key aspects that require continuous monitoring.

## 8. Conclusions

The integration of gut microbiota research into biomimetics has unveiled transformative opportunities to address complex challenges in healthcare, biotechnology, and environmental sustainability. By emulating the dynamic principles of microbial ecosystems—such as resilience, self-regulation, and intercellular communication—biomimetics provides a robust framework for advancing personalized medicine, bioinspired technologies, and sustainable solutions. This review has highlighted key innovations, from the development of artificial microbiomes and precision therapies to the creation of biosensors and environmental bioremediation systems. Despite these advancements, significant challenges remain, including the replication of microbial complexity in artificial environments, ethical considerations surrounding the use of genetically engineered microbes, and the equitable accessibility of emerging therapies. Addressing these issues requires a multidisciplinary approach that integrates advanced tools, such as artificial intelligence and omics technologies, with ethical frameworks and public education initiatives. Interdisciplinary collaboration will be vital to overcome these barriers and ensure that microbiota-based solutions are both innovative and inclusive.

The future of microbiota biomimetics lies in its ability to expand beyond clinical applications, offering solutions for global health and environmental issues. By harnessing the principles of microbial ecosystems, researchers can create adaptive, efficient, and sustainable technologies that benefit both human health and ecological balance. As this field evolves, sustained investment in research, regulation, and education will be essential to fully realize the transformative potential of microbiota biomimetics. By aligning scientific innovation with ethical responsibility, microbiota biomimetics stands poised to revolutionize medicine and redefine the boundaries of sustainable healthcare.

## Data Availability

No new data were created or analyzed in this study.
